# Effects of Aerobic Exercise on Metabolic Syndrome, Cardiorespiratory Fitness, and Symptoms in Schizophrenia Include Decreased Mortality

**DOI:** 10.3389/fpsyt.2018.00690

**Published:** 2018-12-21

**Authors:** Andrea Schmitt, Isabel Maurus, Moritz J. Rossner, Astrid Röh, Moritz Lembeck, Martina von Wilmsdorff, Shun Takahashi, Boris Rauchmann, Daniel Keeser, Alkomiet Hasan, Berend Malchow, Peter Falkai

**Affiliations:** ^1^Department of Psychiatry and Psychotherapy, University Hospital, LMU Munich, Munich, Germany; ^2^Laboratory of Neuroscience (LIM27), Institute of Psychiatry, University of São Paulo, São Paulo, Brazil; ^3^Department of Psychiatry and Psychotherapy, Medical Faculty, Heinrich-Heine-University, Düsseldorf, Germany; ^4^Department of Neuropsychiatry, Wakayama Medical University, Wakayama, Japan; ^5^Department of Radiology, University Hospital, LMU Munich, Munich, Germany; ^6^Department of Psychiatry and Psychotherapy, University Hospital Jena, Jena, Germany

**Keywords:** aerobic exercise, endurance training, high-intensity interval training, metabolic syndrome, mortality, schizophrenia, cognition, positive and negative symptoms

## Abstract

Schizophrenia is a severe psychiatric disorder with a lifetime prevalence of about 1%. People with schizophrenia have a 4-fold higher prevalence of metabolic syndrome than the general population, mainly because of antipsychotic treatment but perhaps also because of decreased physical activity. Metabolic syndrome is a risk factor for cardiovascular diseases, and the risk of these diseases is 2- to 3-fold higher in schizophrenia patients than in the general population. The suicide risk is also higher in schizophrenia, partly as a result of depression, positive, and cognitive symptoms of the disease. The higher suicide rate and higher rate of cardiac mortality, a consequence of the increased prevalance of cardiovascular diseases, contribute to the reduced life expectancy, which is up to 20 years lower than in the general population. Regular physical activity, especially in combination with psychosocial and dietary interventions, can improve parameters of the metabolic syndrome and cardiorespiratory fitness. Furthermore, aerobic exercise has been shown to improve cognitive deficits; total symptom severity, including positive and negative symptoms; depression; quality of life; and global functioning. High-intensity interval endurance training is a feasible and effective way to improve cardiorespiratory fitness and metabolic parameters and has been established as such in somatic disorders. It may have more beneficial effects on the metabolic state than more moderate and continuous endurance training methods, but to date it has not been investigated in schizophrenia patients in controlled, randomized trials. This review discusses physical training methods to improve cardiorespiratory fitness and reduce metabolic syndrome risk factors and symptoms in schizophrenia patients. The results of studies and future high-quality clinical trials are expected to lead to the development of an evidence-based physical training program for patients that includes practical recommendations, such as the optimal length and type of aerobic exercise programs and the ideal combination of exercise, psychoeducation, and individual weight management sessions.

## Introduction

Schizophrenia is a severe neuropsychiatric disease that affects ~1% of the population (1, 2). It strikes young adults between 20 and 30 years of age (3), and symptoms persist throughout adult life in 30–50% of affected patients (4, 5). The disease leads to impaired social functioning; for example, only 20% of patients are able to have a job on the primary market, and only about 30% have a stable relationship (5). Because of the high hospitalization rates and high levels of both disease-related incapacity to work and early retirement, schizophrenia-related costs exceed even those of widespread diseases such as cardiovascular diseases (CVD) (6). According to the WHO report on the global burden of disease, schizophrenia is one of the most common conditions associated with a high rate of years lived with disability (YLD), one of the leading causes of disease burden (7), and among the 10 most costly illnesses worldwide (8).

## Cognitive Impairment and Underlying Neurobiological Pathways

Cognitive impairment is a frequent core feature of schizophrenia (9), in addition to positive and negative symptoms, and is related to the reported volume loss of the hippocampus, a region central to memory, learning, and cognitive integration (10). Cognitive deficits and negative symptoms are the most important predictors for poor social and functional outcome and are major contributors to disability (11). Cognitive deficits are present in domains such as executive function, episodic memory, attention, and social cognition (11–13), functions that are particularly dependent on the hippocampus and prefrontal cortex (14). In schizophrenia, neurodevelopmental disturbances in vulnerable periods of brain development are thought to lead to hippocampal-prefrontal pathway deficits, resulting in the onset of disease symptoms in young adulthood (15). Meta-analyses of structural magnetic resonance imaging (sMRI) studies revealed gray matter volume reduction of 5–10% in the prefrontal cortex and hippocampus in schizophrenia patients (16). In the polymorph layer of the dentate gyrus (CA4) of the hippocampus, decreased numbers of oligodendrocytes (17, 18)—an indicator of disturbed myelination—have been detected in schizophrenia. A meta-analysis of diffusion-tensor imaging (DTI) studies in schizophrenia revealed decreased fractional anisotropy in white matter tracts interconnecting the prefrontal cortex and hippocampus (19); such white matter disruption is known to play a crucial role in cognition and psychopathology (20). Of interest in this context is that schizophrenia patients with overweight and obesity showed disturbed white matter integrity, with lower fractional anisotropy than normal-weight schizophrenia patients (21).

Several environmental factors, such as psychosocial stress associated e.g., with an urban lifestyle or childhood trauma, interact with genetic factors to increase the risk of a chronic disease course (22). The prefrontal cortex and hippocampus are central to cognitive processing and are also involved in the regulation of the neuroendocrine control of stress hormone secretion, including glucocorticoids (23). Whereas, acute stress can increase fear-associated memory, chronic stress with cortisol-based stress reactivity levels has a negative impact on spatial-reference memory and cognitive flexibility, induces hippocampal volume loss, adversely alters dendritic morphology and reduces adult neurogenesis and synaptic plasticity (23). Chronic stress during the pubertal period up to young adulthood, when synaptic pruning and oligodendrocyte-related myelination take place, has been shown to induce symptoms of schizophrenia (24).

## Mortality in Schizophrenia

Schizophrenia affects daily life and subjective well-being. Low physical activity, impaired physical health, and reduced activities of daily living (25, 26) are just some aspects of the disease. Compared with the general population, individuals with schizophrenia have a 12-fold higher mortality from all external causes (27), which can largely be explained by the high incidence of somatic comorbidities; unhealthy lifestyles, such as high rates of cigarette smoking and low physical activity; and increased rate of suicides (26, 28, 29). Estimates indicate that together these health-related risk factors and suicides reduce life expectancy by nearly 10–20 years compared with the general population (30, 31). A meta-analysis of data from 29 countries on six continents found that mortality was significantly higher among people with mental disorders and that, in 65 studies, the highest mortality rate (relative risk 2.54, 95% CI 2.35–2.75) was among patients with psychosis (31). The relative risk for natural causes, such as CVD, was 1.80 (95% CI 1.71–1.88), but that for unnatural causes, such as suicides, was even higher (7.22, 95% CI 6.43–8.12). Cardiovascular risk was higher in schizophrenia patients than in patients with depressive disorder or multiple psychiatric diagnoses (32). People with severe mental illness, including schizophrenia, had a higher risk of developing coronary heart disease than controls (adjusted hazard ratio 1.54; 95% CI 1.30–1.82) and a higher rate of autonomic nervous system dysfunction, including diminished heart rate variability, hypertension, alterations of the QT interval, and lipid pattern abnormalities (33). During the year after the first diagnosis of psychosis, a study found that the relative risk for all-cause mortality was 54.6 (95% CI 41.3–68.0) per 10,000, whereby the highest relative risk of death was found for self-inflicted injury or poisoning; during this period, the relative risk of death due to heart disease or diabetes did not differ between the group of people with a psychotic disorder and the general outpatient group (34).

In schizophrenia patients, suicidal thoughts and suicide planning and attempts were significantly associated with completed suicide in the following year (35). In first-episode patients, more symptoms of depression, longer duration of untreated psychosis, and positive symptoms, such as hallucinations and delusions, were found to increase the odds of experiencing suicidal ideation (36). Another study found that the severity of negative symptoms was lower in schizophrenia patients who attempted suicide (37). With respect to cognitive performance, decreased global cognitive functioning and visual memory predicted suicidal behavior in non-affective psychosis (38). In a meta-analysis and meta-regression analysis in over 80,000 patients with schizophrenia, depressive symptoms, the Positive and Negative Symptom Scale (PANSS) general score, and the number of hospitalizations were higher in patients with suicidal ideation (39). A history of alcohol use, family history of psychiatric illness, physical comorbidity, history of depression, and depressive symptoms were associated with suicide attempts, whereas poor adherence to treatment, hopelessness, higher intelligence quotient, history of attempted suicide, and being male were most consistently associated with completed suicide (39).

This qualitative review will provide a current overview of clinical studies aimed at reducing the socioeconomic burden of schizophrenia and mortality by addressing metabolic risk factors and symptom severity in schizophrenia patients.

## The Metabolic Syndrome in Schizophrenia

The International Diabetes Federation defines the metabolic syndrome as a combination of increased waist circumference (a mandatory feature) and two of the following criteria: elevated triglycerides, high blood pressure, elevated fasting glucose, and low high-density lipoprotein (HDL) cholesterol (Table 1) (40). Metabolic syndrome is defined slightly differently by the Adult Treatment Panel III (ATP III) of the National Cholesterol Education Program (41) and the adapted Adult Treatment Panel III (ATP III-A) of the American Heart Association (42), both of which require 3 of 5 criteria to be fulfilled (Table 2). In the general population, the metabolic syndrome is associated with a 4-fold increased relative risk to develop type 2 diabetes mellitus (43) and a 2-fold increased relative risk to develop CVD, such as stroke and coronary heart disease (44). Schizophrenia patients have a higher prevalence of metabolic syndrome than the general population and a 2- to 3-fold increased risk for CVD, resulting in increased cardiac mortality (30, 45). Metabolic syndrome was present in 37.3% of schizophrenia patients treated with second-generation antipsychotics and associated with an increased 10-year risk of coronary heart disease (risk ratio 2.18, 95% CI 1.88–2.48) in both male and female patients (risk ratio 1.94, 95% CI 1.65–2.23). Among the criteria for metabolic syndrome, triglyceride levels and waist circumference were significantly associated with the 10-year risk of coronary heart disease events (46). A meta-analysis found that the overall rate of metabolic syndrome in schizophrenia patients was 32.5% (95% CI 30.1–35.0%) and showed only minor differences between treatment settings (inpatient vs. outpatient), country of origin, and gender. Duration of illness and older age had the strongest influence. Among the criteria for metabolic syndrome, waist circumference was the strongest predictor (47). Additionally, the prevalence of metabolic syndrome was higher in patients with negative symptoms, which are associated with a sedentary lifestyle and lack of physical activity (48). Furthermore, in schizophrenia patients metabolic syndrome was significantly associated with cognitive impairment and was found to contribute to cognitive deficits throughout the course of the disease (49).

**Table 1:** International Diabetes Federation criteria for metabolic syndrome.

**Measure**	**Threshold (Waist circumference plus 2 other measures required)**
Elevated waist circumference	
Men	≥94 cm
Women	≥80 cm
Elevated triglycerides	≥150 mg/dl
Elevated blood pressure^*^	≥130 mm Hg systolic blood pressure or ≥85 mm Hg diastolic blood pressure
Reduced high-density lipoprotein cholesterol	
Men	< 40 mg/dl
Women	< 50 mg/dl
Elevated fasting glucose^**^	≥100 mg/dl

* *or treated with antihypertensive medication*;

** *or treated with insulin or hypoglycaemic medication*.

**Table 2:** Adult Treatment Panel III and III-A criteria for metabolic syndrome.

**Measure**	**Clinical criteria ATP III (3 of 5 required)**	**Threshold ATP III-A(3 of 5 required)**
Elevated waist circumference		
Men	≥102 cm	≥102 cm
Women	≥88 cm	≥88 cm
Elevated triglycerides	≥150 mg/dl	≥150 mg/dl
Elevated blood pressure	≥130 mm Hg systolic blood pressure or ≥85 mm Hg diastolic blood pressure	≥130 mm Hg systolic blood pressure or ≥85 mm Hg diastolic blood pressure
Reduced high-density lipoprotein cholesterol		
Men	< 40 mg/dl	< 40 mg/dl
Women	< 50 mg/dl	< 50 mg/dl
Elevated fasting glucose	≥110 mg/dl	≥100 mg/dl

One study found that the risk of metabolic syndrome was elevated in all patients with severe mental illness (32.6%, 95% CI 30.8–34.3%) and did not differ between patients with schizophrenia and those with bipolar disorder or between patients with bipolar disorder and those with major depression (45). In a meta-analysis of first-episode schizophrenia patients, the rate of metabolic syndrome was only 9.9%; the rate of overweight was 22%; hypertriglyceridemia, 19.6%; low HDL, 21.9%; hyperglycaemia, 6.4%; high blood pressure 24.3%; and smoking, 46.8% (50). This indicates that the cardiovascular risk is lower in first-episode than in multi-episode schizophrenia patients. Longer duration of illness is also predictive for longer treatment with antipsychotics, and treatment with second-generation antipsychotics in particular is a risk factor for developing metabolic syndrome (see below).

## Antipsychotic Treatment and Metabolic Syndrome

Despite reducing positive symptoms, first- and second-generation antipsychotics have demonstrated only poor or no efficacy in improving cognitive deficits and negative symptoms in schizophrenia (51, 52). Depending on the dose, users of typical and atypical antipsychotics had higher rates of sudden cardiac death than non-users (adjusted incidence rate 1.99, 95% CI 1.68–2.34) (53). Furthermore, atypical antipsychotics are known to prolong the QTc interval and increase resting heart rate, thereby affecting autonomic neurocardiac function (54). However, a decrease in heart rate variability has also been shown in unmedicated schizophrenia patients and is a cardiac risk factor, together with low physical fitness (55). A meta-analysis showed that second-generation antipsychotics have fewer extrapyramidal side effects than first-generation drugs, such as haloperidol (52). Many of the most effective second-generation antipsychotics, including olanzapine, clozapine, and risperidone (52, 56), however, are associated with substantial weight gain and sedation (52), leading to increased rates of the metabolic syndrome. In unmedicated and first-episode schizophrenia patients, the overall rate of the metabolic syndrome was only about 10%, the rate of diabetes was only 1–2%, and the rate of overweight was 22–26%. Therefore, the cardiovascular risk can be assumed to be higher in multi-episode patients with a long treatment history (50). A meta-analysis found that patients with severe mental illness who were treated with any antipsychotic had a significantly higher risk of metabolic syndrome than antipsychotic-naïve individuals. The risk was higher with olanzapine and clozapine than with other antipsychotics, especially aripirazole, and higher in patients on polypharmacotherapy than in those on monotherapy (45). Another meta-analysis also found the highest rates of metabolic syndrome in patients treated with clozapine (51.9%) and the lowest rates in unmedicated patients (20.2%) (47). One study found a positive association between a serotonin receptor gene (HTR2C) polymorphism and metabolic syndrome in patients treated with olanzapine, clozapine, and risperidone and that genetic factors may influence the prevalence of the metabolic syndrome in schizophrenia (57).

In summary, lifestyle changes can be assumed to be warranted, especially in multi-episode patients on long-term antipsychotic treatment. To reduce the risk for metabolic syndrome, subsequent CVD, and mortality, add-on therapeutic interventions aimed at improving symptoms of the disease, such as depression or negative symptoms; reducing the smoking rate; and increasing physical activity are needed (58).

## Physical Activity in Schizophrenia

A worldwide analysis of adverse health conditions in the general population estimated that physical inactivity causes 6% of the burden of disease from coronary heart disease; 7%, from type 2 diabetes; and 10%, from breast cancer and colon cancer. Overall, inactivity causes 9% of premature mortality (59). The health benefits of physical activity in adults include reduced rates of metabolic syndrome, coronary heart disease, high blood pressure, type 2 diabetes, stroke, depression, and cancer. Additionally, there is strong evidence for increased cardiorespiratory fitness, healthier body mass and composition, and improved cognitive functioning (59). The high prevalence of the metabolic syndrome in schizophrenia may be a result of the disease itself and treatment with antipsychotics (see above) or of sedentary behavior and low physical activity and aerobic fitness, which play an important role in this patient group (60, 61), or a combination of these factors. Studies have repeatedly shown that schizophrenia patients show reduced cardiovascular fitness and physical activity (62). In a meta-analysis of 13 studies, sedentary behavior measured in hours per day was significantly higher in schizophrenia patients (hedges g = 1.13, 95% CI 1.47–4.1) than in healthy controls (63). According to a meta-analysis, schizophrenia patients engage in less moderate (hedges g = −0.45, 95% CI −0.79 to −0.1) and vigorous physical activity (g = −0.4, 95% CI −0.60 to −0.18) than healthy controls, and depressive symptoms and older age are associated with less vigorous physical activity (64). A meta-analysis of 212 schizophrenia patients and 132 healthy individuals confirmed that physical activity was lower in the patient group and showed that decreased physical activity was correlated with impaired quality of life and social functioning, increased social withdrawal, and lower motivation and employment rates (65). Furthermore, low physical fitness was associated with illness duration, smoking, the presence of the metabolic syndrome, and more severe negative, depressive, and cognitive symptoms (26). In schizophrenia patients, the duration of physical activity was negatively correlated with waist circumference and body mass index, while food intake variables correlated with HDL cholesterol and triglyceride levels (66). A systematic review found that lifestyle interventions with psychoeducation, diet, and recommendations on physical activity were associated with significant weight reduction, reduced body mass index, decreased waist circumference, and lower blood glucose levels (67).

## Requirements for Aerobic Exercise Studies in Schizophrenia

Compared with the healthy population, patients with schizophrenia have specific characteristics that decrease their motivation to perform physical activity; these characteristics include sedation related to antipsychotic treatment; schizophrenia symptoms, including anxiety and depression; a lower level of education; little experience with exercise; social withdrawal; and negative symptoms (68, 69). Therefore, it is essential that aerobic exercise interventions aimed at improving cardiovascular fitness are supervised by a sports scientist to increase patients' motivation to participate (70). Schizophrenia patients and healthy controls showed comparable adaptations to endurance training, as assessed by physical working capacity and maximal achieved power, but differences were found in changes of performance at a given lactate concentration (70). Under supervision by a sports scientist, endurance training was feasible and effective in both groups (70). The first studies to investigate aerobic exercise in schizophrenia patients had many methodological limitations (71). Major concerns included missing healthy or patient control groups or both, inadequate sample sizes, and non-randomized or non-controlled designs (72–74). In later endurance training studies, a minimum of 30 min per training session and at least three sessions per week were recommended in schizophrenia patients (75). According to the criteria of the American College of Sports Medicine, in healthy individuals 150 min of moderate training per week are necessary to improve cardiorespiratory fitness (76). A recent cross-sectional study examined the exercise behavior and mental health of 1,237,194 people aged 18 years or older in the USA and found that individuals who exercised had 43.2% fewer days of poor mental health in the past month and a lower mental health burden than the non-exercising groups. Interestingly, the largest effects were seen for popular team sports, cycling, and aerobic and gym activities and for durations of 45 min and frequencies of three to five times per week (77).

## Effects of Aerobic Exercise Interventions on the Metabolic Syndrome

New add-on treatment options, such as aerobic exercise, are needed to reduce the risk of cardiometabolic diseases in schizophrenia. However, results of intervention studies examining the effects endurance training on parameters of the metabolic syndrome in schizophrenia patients were mainly negative. In a combined Weight Watchers intervention, exercise had no effects on weight loss (78). A randomized controlled trial in schizophrenia patients consisting of 2 h of aerobic exercise per week over a period of 6 months had no effects on body mass index, body fat percentage, or factors of the metabolic syndrome (79). A meta-analysis of aerobic exercise interventions in patients with bipolar or schizophrenia spectrum disorders found no effects of aerobic exercise on body weight or body mass index (80), and another meta-analysis also reported no effects of aerobic exercise on body mass index in schizophrenia patients (81). In contrast, in a small sample of schizophrenia patients aerobic exercise improved physical activity, blood pressure, and body composition (82). In a randomized study of an intervention comprising aerobic group exercise and individual weight management sessions in obese or overweight patients with schizophrenia, schizoaffective disorder, bipolar disorder, and major depression, weight loss in the intervention group increased progressively over the 18-month study period and differed from the control group (83). In summary, findings to date indicate that isolated exercise interventions are unlikely to induce weight loss in patients with schizophrenia (84). However, adding psychosocial interventions or diet to aerobic exercise programs seem to be a promising approach to reduce body weight. Increased physical activity (pedometer walking) plus motivational interviewing reduced body weight in obese schizophrenia patients after 12 weeks (85). In obese schizophrenia patients, a 3-month lifestyle intervention comprising psychosocial treatment, behavior therapy, and aerobic exercise reduced waist circumference, body weight, and body mass index but did not affect blood lipids or glucose levels (86). In a small study in multi-episode schizophrenia patients, an aerobic exercise program consisting of three 30-min sessions per week over a period of 24 weeks significantly reduced weight and body mass index (73). In a personalized diet and exercise program (3 exercise sessions per week) in 106 schizophrenia patients treated with antipsychotics, the months of participation correlated with weight loss (87) (Table 3).

**Table 3:** Effects of continuous endurance training on metabolic risk factor and symptoms of the disease in patients with schizophrenia.

**Study**	**Participants**	**Training methods**	**Effects on cardio-respiratory fitness**	**Effects on metabolic partameters**	**Effects on symptoms**
Dodd et al. (73)	8 chronic schizophrenia patients	24 weeks aerobic exercise program (treadmill, bicylcle, walking)		Body weight **↓**Body mass index **↓**	
Methapatara and Srisurapanont (85)	64 schizophrenia patients with body mass index of 23 kg/m^2^ or more	12 weeks randomized controlled trial with pedometer walking plus 1 week motivational inertviewing program vs. usual care		Body weight **↓**Waist circumference **↓**Body mass index **↓**	
Pajonk et al. (88)	16 chronic schizophrenia patients 8 healthy controls	3 months randomized controlled trial with cycling vs. table football	VO_2max_ **↑** W_peak_ **↑**		Short-term verbal memory **↑** Total symptoms **↓**
Scheewe et al. (89)	63 schizophrenia patients, 55 healthy controls	6 months randomized controlled trial with cardiovascular aerobic exercise and muscle strength exercises vs. occupational therapy	VO_2peak_ **↑** W_peak_ **↑**		
Daumit et al. (83)	291 overweight or obese patients with schizophrenia (58%), bipolar disorder (22%) or major depression (12%)	18 months group exercise sessions plus weight management sessions vs. standard information on nutrition and physical activity		Body weight **↓**	
Scheewe et al. (79)	63 schizophrenia patients	6 months randomized controlled trial with cardiovascular aerobic exercise and muscle strength exercises vs. occupational therapy	W_peak_ **↑**	Body mass index -Waist circumference -	Depressive symptoms **↓** Total symptoms **↓** Positive symptoms **↓** Disorganization **↓** Excitement **↓** Emotional distress **↓**
Bredin et al. (82)	13 schizophrenia patients	12 weeks aerobic exercise (cycling, treadmill, elliptical training)	VO_2peak_ **↑** W_peak_ **↑**	Body weight **↓**Waist circumference **↓**	
Kuo et al. (86)	33 obese schizophrenia patients 30 healthy controls	10 weeks aerobic exercise, lifrestyle modification, psychosocial treatment, behavior therapy		Body weight **↓**Waist circumference **↓**Body mass index **↓**	
Malchow et al. (90)	43 multi-episode schizophrenia patients, 22 healthy controls	3 months aerobic endurance training (cycling) plus cognitive remediation vs. table football plus cognitive remediation			Global functioning **↑** Social adjustment **↑** Negative symptoms **↓** Short-term verbal memory **↑** Cognitive flexibility **↑**
Amiaz et al. (87)	106 schizophrenia patients	9 months fitness and diet program		Body weight **↓**Waist circumference **↓**Body mass index **↓**Body fat percentage **↓**	
Armstrong et al. (91)	33 patients with schizophrenia	12 weeks randomized controlled trial with aerobic exercise vs. treatment as usual	VO_2peak_ **↑** W_peak_ **↑**	Body weight -Body mass index -	
Jerome et al. (92)	291 overweight or obese patients with schizophrenia (58%), bipolar disorder (22%) or major depression (12%)	18 months group exercise sessions plus weight management sessions vs. standard information on nutrition and physical activity	Heart rate response **↓**		
Firth et al. (93)	38 patients with first-episode schizophrenia	10 weeks individualized aerobic exercise vs. treatment as usual			Positive symptoms **↓** Negative symptoms **↓** Psychosocial functioning **↑** Short-term verbal memory **↑**

*VO_2_peak, VO_2_max,highest oxygen intake; W_peak_, peak work rate/power output*.

Because of the need to improve the efficacy of aerobic exercise, higher intensity training, such as high-intensity interval training (HIIT), has been used in obese individuals. HIIT is a promising new method of intensified endurance training. A 12-week HIIT significantly improved metabolic parameters, such as waist circumference, body mass, fasting glucose, HDL cholesterol, and blood pressure, in mentally healthy patients with cardiometabolic risk factors and obesity (94–98). HIT has the highest potential to reduce visceral adipose tissue in obese individuals (99). Compared with continuous training, HIIT significantly reduced insulin resistance, HbA1c, and body weight in healthy adults and reduced fasting glucose in participants at risk of type 2 diabetes (100). However, a recent meta-analysis found no difference between HIIT and moderate-intensity continuous training for body fat reduction (101). This finding was supported by a meta-analysis of 12-month interventions in obese adults, which showed weight loss but found no difference between HIIT and moderate-intensity exercise (102). Another meta-analysis showed that HIIT performed as cycling or running significantly reduced abdominal and visceral fat mass but that running was more effective than cycling (103). In a group of obese men, HIIT significantly improved insulin sensitivity and muscle mitochondrial content (increased muscle mitochondrial content is assumed to be a basic mechanism of effect of HIIT) compared with continuous aerobic training (104). To date, only two studies have applied HIIT interventions in patients with schizophrenia. One evaluated a 14-week program of 40 min HIIT twice a week in first-episode patients and showed a significant decrease in waist circumference and heart rate (105). The other studied an 8-week HIIT program comprising 25-min sessions three times a week in multi-episode patients and showed reduced body weight, body mass index, and resting heart rate (106) (Table 4). Overall, HIIT has the potential to improve parameters of the metabolic syndrome in patients with schizophrenia.

**Table 4:** Effects of high-intensity interval training on metabolic risk factors and symptoms of the disease in patients with schizophrenia.

**Study**	**Participants**	**Training methods**	**Effects on cardio-respiratory fitness**	**Effects on metabolic partameters**	**Effects on symptoms**
Heggelund et al. (107)	25 inpatients	8 weeks HIIT vs. playing computer games	VO_2peak_ **↑**		No change in positive, negative symptoms or depression
Abdel-Baki et al. (105)	25 first-episode patients	14 week HIIT	VO_2max_ **↑** Resting heart rate **↓**	Waist circumference **↓**Body weight -Body mass index	
Heggelund et al. (108)	20 patients with schizophrenia, 13 patients with depression, 20 healthy individuals	1 day HIIT			Positive affect in all participants Patients with depression and schizophrenia had reduced distress and state anxiety
Herbsleb et al. (109)	Case report in one patient with schizophrenia	6 weeks HIIT vs. CET	Resting heart rate **↓** Heart rate variability **↑** VO_2peak_ **↑**	Body weight -Body mass index –Body fat percentage -	
Wu et al. (106)	20 patients with chronic schizophrenia	8 weeks HIIT	Resting heart rate **↓** Pulse pressure **↓** Mean arterial pressure **↑** Diastolic blood pressure **↑**	Body weight **↓**Body mass index **↓**	Negative symptoms improved. General psychopathology improved. Depression and anxiety improved

*HIIT, high intensity interval training; CET, continuous endurance training; VO_2_peak, VO_2_max, highest oxygen intake*.

## Effects of Aerobic Exercise Interventions on Cardiorespiratory Fitness

In the general population, improving cardiorespiratory fitness is a key factor in preventing CVD and mortality (110, 111). Cardiorespiratory fitness can be measured by the highest oxygen intake (referred to as VO_2max_ or VO_2peak_) and peak work rate/power output (W_peak_). Aerobic exercise interventions seeking to improve cardiorespiratory fitness can prevent CVD and the associated mortality (112). In a randomized study in obese and overweight patients with severe mental illness, including schizophrenia, schizoaffective disorder, bipolar disorder, and major depression, participation in group exercise classes was associated with improved short- and long-term cardiorespiratory fitness, as indicated by a lower heart rate response (92). Significant increases in VO_2max_, as a measure of aerobic capacity, and power output (W_peak_) have been reported in schizophrenia patients after continuous exercise training compared with control conditions, such as occupational therapy or table soccer (73, 79, 82, 88, 89, 91, 113) (Table 3). In a meta-analysis, cardiorespiratory fitness was improved in schizophrenia patients after aerobic exercise (g = 0.43, 95% CI 0.05–0.82). Furthermore, in schizophrenia patients improved cardiorespiratory fitness was correlated with an increased volume of the hippocampus (88, 114), with decreased ventricular and increased cerebral gray matter volume, and with thickening in the frontal, temporal, and cingulate cortex of the left hemisphere (113).

In a meta-analysis of HIIT vs. moderate-intensity continuous training, HIIT was more likely to increase VO_2peak_ in adults with coronary heart disease, hypertension, metabolic syndrome, and obesity (115). In obese adults with hypertension, HIIT and continuous training both improved cardiorespiratory fitness, whereby an exercise intervention lasting at least 12 or 16 weeks was needed to achieve these effects (116). In schizophrenia patients, HIIT improved VO_2peak_ by 12% compared with playing computer games (107), and in a case report of a patient with schizophrenia HIIT was more effective than moderate continuous training in increasing heart rate variability and reduing resting heart rate (109). A decrease in resting heart rate and 38% increase in VO_2max_ was detected after a 14-week HIT program in schizophrenia patients (105). In summary, aerobic exercise is capable of improving cardiorespiratory fitness and thereby reducing risk factors for CVD and associated mortality (Table 4).

## The Impact of Aerobic Exercise on Schizophrenia Symptoms and Cognition

Reduction of symptoms may help to decrease suicidality in patients with schizophrenia (see above), and there is evidence that aerobic exercise interventions can improve schizophrenia symptoms and cognitive deficits. Published data from our group show that a structured endurance training programme is feasible in multi-episode schizophrenia patients (70). Previously, we demonstrated that 3 x 30 min aerobic exercise per week alleviated negative symptoms, significantly improved short-term memory and increased hippocampal volumes in patients with schizophrenia (88). Furthermore, when we added cognitive training to aerobic exercise from week 6 up to 3 months negative symptoms and working memory improved (90). Importantly, in 45% of the multi-episode schizophrenia patients who performed aerobic exercise the Global Assessment of Functioning (GAF) score improved by about 20% from baseline to month 3 (90); thus, this study showed that aerobic exercise as add-on therapy fosters patients' capacity to improve in multi-epsode schizophrenia. In this study, we did not replicate the increase of hippocampal volume after aerobic exercise, although we did detect increased volume of the left temporal cortex (117). In a randomized clinical trial, a 6-month structured aerobic exercise intervention reduced total symptoms of schizophrenia, positive symptoms, disorganization, excitement, emotional distress, symptoms of depression, and need of care significantly more than occupational therapy (79). In first-episode patients, an individualized exercise training program revealed improvement of negative and positive symptoms, psychosocial functioning and verbal short-term memory (93).

The beneficial effects of physical exercise on brain function and structure and cognitive performance have been repeatedly reported in healthy individuals [e.g., (118)]. Beyond this, a meta-analysis showed that in schizophrenia patients aerobic exercise improves negative, positive, and depressive symptoms and global functioning, as measured by the GAF (119). Another meta-analysis focusing on cognition demonstrated improved global cognition, working memory, social cognition, and attention after aerobic exercise in schizophrenia patients (120). Findings from the literature are contradictory, but a meta-analysis of exercise interventions in schizophrenia indicated that symptoms improve with a higher intensity of training (81). Symptom improvement was prominent in interventions of 90 min of moderate exercise per week (81). In healthy adults, elderly people, or individuals with obesity, HIIT improved executive function and verbal and short-term memory (95, 121). A meta-analysis in healthy normal-weight and obese individuals showed that HIIT training improved affective psychological responses (122). In a first study in a small group of patients with different diagnoses that included only some schizophrenia patients, 8 weeks of HIIT did not decrease psychopathological symptoms, but it did have positive effects on physical fitness and anxiety (107, 108). In 18 patients with schizophrenia, an 8-week HIIT program significantly improved negative symptoms, general psychopathology, depression, and anxiety (106) (Table 4).

## Conclusion

In summary, add-on therapy of endurance training combined with psychosocial interventions or diet may improve symptoms of the metabolic syndrome in schizophrenia patients, thereby reducing the prevalence of CVD and mortality. It has been shown that aerobic exercise programs are feasible in populations with severe mental illness and are accepted by schizophrenia patients who have weight gain and reduced physical fitness. However, specific complicating characteristics of patients with schizophrenia in comparison to the healthy population include fatigue and sedation (e.g., due to antipsychotic treatment), symptoms of the disease, a high level of anxiety and depression, antipsychotic-induced weight gain, a lower level of education, little experience with sport, and a lack of motivation for physical activity in case of negative symptoms (70). To increase patients' motivation to adhere to training sessions, a sports scientist must provide supervision, which in turn increases the cost of otherwise cheap training methods, such as biking and walking. Nevertheless, aerobic exercise programs still have a good cost-benefit ratio when one considers the high socioeconomic costs of metabolic risk factors in these patients. To date, physical exercise has no known serious side effects or safety issues that might pose any risk to the patients. Aerobic exercise can significantly contribute to improving symptoms of the disease, including cognitive deficits and psychosocial functioning. In addition, it may have positive effects on the residual symptoms that are known to be treatment resistant even after long-term therapy with antipsychotics; however, treatment recommendations will be given when the optimal dose and duration of the intervention has been found in randomized clinical trials. The reversibility of structural alterations in the brain and improvement of symptoms suggest that aerobic exercise may induce a regenerative process in patients with schizophrenia (123). Finally, HIIT can be hypothesized to have greater potential than conventional, aerobic endurance training to improve cognitive deficits, overall symptoms, and metabolic parameters in schizophrenia patients. Future studies should investigate the effects of HIIT on neuroplastic changes in the brain. Furthermore, treatment recommendations should include aerobic exercise in multimodal therapy regimes. The German S3 guideline “Schizophrenia” (124) and the NICE guideline (125) recommend exercise programs for patients with schizophrenia and weight gain. However, positive effects of aerobic exercise may be limited to the training periods (90), and long-term improvement of metabolic risk factors may require continuation of aerobic training in certified sports clubs. Overall, further high quality clinical trials are needed before statements can be made about the optimal length and type of aerobic exercise programs for routine clinical care.

## Author Contributions

PF, BM, AS, and IM designed this manuscript. ST, DK, BR, AS, BM, IM, MR, AR, AH, ML, MvW, and PF managed the literature searches, interpreted the data and prepared the manuscript. All authors contributed to and approved the final manuscript and reviewed it critically for important intellectual content.

### Conflict of Interest Statement

AH received funding from Lundbeck, Janssen-Cilag, and Pfizer and a paid speakership from Desitin, Otsuka, and Lundbeck and was a member of a Roche advisory board. PF has been an honorary speaker for AstraZeneca, Bristol Myers Squibb, Lilly, Essex, GE Healthcare, GlaxoSmithKline, Janssen Cilag, Lundbeck, Otsuka, Pfizer, Servier, and Takeda and a member of the advisory boards of Janssen-Cilag, AstraZeneca, Lilly, and Lundbeck. AS was an honorary speaker for TAD Pharma and Roche and a member of Roche advisory boards. The remaining authors declare that the research was conducted in the absence of any commercial or financial relationships that could be construed as a potential conflict of interest.
